# Mixed-method evaluation of the Fairer Futures Fund: a £22.2 m public health initiative designed to reduce health inequalities in Birmingham and Solihull (UK)

**DOI:** 10.3389/fpubh.2025.1658646

**Published:** 2025-09-29

**Authors:** Ian Litchfield, Andy Knight, Rebecca Howell-Jones, David Miller, Zoe Sweeney, Mike Walsh, Lorraine Harper

**Affiliations:** ^1^Department of Applied Health Sciences, University of Birmingham, Birmingham, United Kingdom; ^2^Birmingham Health Partners, Birmingham, United Kingdom; ^3^Birmingham City Council, Birmingham, United Kingdom; ^4^Heart of England Community Foundation Trust, Coventry, United Kingdom; ^5^University Hospitals Birmingham NHS Foundation Trust, Birmingham, United Kingdom

**Keywords:** community participation, public health, integrated care, health inequalities, Preventive care

## Abstract

**Introduction:**

Birmingham and Solihull Integrated Care System created the £22.2 million Fairer Futures Fund to support community-centred collaborative innovation to address long-standing health inequalities in the region. The FFF supports the development of productive and sustainable partnerships between communities, the voluntary sector, and health and social care organisations to deliver three broad categories of projects: multiple small community-located projects; large-scale ‘partnership’ projects led by collaborative partnerships, and a series of system-wide projects intended to support infrastructural interventions across organisations in support of more equitable care.

**Methods and analysis:**

The work consists of a mixed-method evaluation conducted over three work packages: One, a qualitative exploration of the factors influencing the design and development of the Fairer Futures Fund programme. Two, a qualitative exploration of the effectiveness of brokered co-design processes and collaborative inter-agency working. Three, a mixed-methods evaluation of all projects, collating and analysing quantitative outputs, contextualised by a qualitative exploration of the experiences of intervention leads and participants. The quantitative data will be summarised using descriptive statistics with the ability of data to be collated and analysed by various shared characteristics across projects using univariable analyses, e.g., paired t-tests (where pre- and post-intervention outcome data are available) and/or multivariable regression analysis to assess the effectiveness of the FFF activities. The qualitative data from across the three work packages will be used in a directed content analysis to populate the Consolidate Framework of Implementation Research.

**Discussion:**

The amount invested, the innovative nature of the funding allocation, and the visibility of the FFF programme warrants this detailed and objective evaluation by experienced researchers and evaluators. To support the success of the FFF, the work will provide both formative and summative findings. Although the precise content and structure of each project is determined by the local communities and collaborating organisations, consistent data collection is supported by the requisite use of a combination of preselected and validated survey tools. This will allow for the collation of larger and comparable data across similar projects. The quantitative data outputs will be contextualised by qualitative data synthesised across work packages using the implementation framework.

## 1 Introduction

Health inequalities continue to persist across the UK, adversely affecting some of the most vulnerable and marginalised members of society including those with learning difficulties, mental health issues, the older adults, and ethnic minorities (1, 2). One area in the UK where these inequalities remain particularly significant is the city of Birmingham and its neighbouring metropolitan borough of Solihull (3). The patient population of 1.3 million is super-diverse, i.e., composed of individuals from diverse ethnic, racial, and cultural backgrounds, with a mix of migration statuses and legal rights; it is also one of the youngest populations in the UK, with high rates of unemployment, and infant mortality (4). Its underserved populations [defined for the purposes of this protocol as those who are economically deprived and/or from ethnic minorities that are engaged less effectively by formal healthcare interventions (5)] continue to have some of the worst health outcomes in the country including for chronic disease, mental health, and perinatal and infant health (6).

The determinants of health inequalities amongst the underserved are complex and multi-factorial and include the impact of localised cultural, environmental, and economic factors (7, 8). Amidst this complexity, multiple attempts at reducing these inequalities, in Birmingham and Solihull and elsewhere nationally and internationally have failed to sustain change, and policy makers, commissioners, and local authorities delivering public health services need more robust evidence to inform the design of grant programmes, interventions and the allocation of funds (9–12). What is more widely understood is that for any such initiatives to be successful then the concerns, needs and experiences of target populations must be effectively accommodated, a process requiring considerable time, support and funding (13–17).

In Birmingham and Solihull (and in common with other regions in England and Wales) health and social care and a range of preventative health initiatives is the responsibility of the local Integrated Care Board (18). In 2024 Birmingham Solihull-Integrated Care Board (BSOL-ICB) created the £22.2 million Fairer Futures Fund (FFF), collectively intended to deliver lasting reductions in health inequalities, including perinatal health, mental health and wellbeing, chronic conditions, and community engagement as set out in BSOL-ICBs 10-year Integrated Health and Care Strategy (19).

Of the total Fairer Futures Fund, £18.2 million was allocated to a grant scheme intended to create cross-sector collaborations and co-produce a range of culturally sensitive interventions to make lasting improvements to health inequalities and shift the focus toward more localised and preventative care (13, 17, 19). The intention is that the funded projects will also contribute to the development and/or delivery of “neighbourhood multi-disciplinary care teams” which are the care model being promoted by the Department of Health (20). (Latterly BSOL-ICB introduced an additional funding stream of circa £3 m as part of FFF to directly support locality delivery of Integrated Neighbourhood Teams as well as Digital and Organisational Development work, though this is beyond the scope of this evaluation).

The FFF comprises seven funding streams which can be usefully summarised within three broad categories:

1. Small-Scale Grants to fund projects of between £10 K and £15 K, initiated by local communities.
2. Large-Scale Grants designed to fund projects of up to £500 K to enable lasting partnerships and co-produced interventions delivered between communities and health and social care organisations.
3. Infrastructure Grants intended to fund projects each of circa £150 K designed to improve infrastructure across BSOL-ICS to enable the delivery of more equitable health and social care services.

The target areas and structure of these three categories of grant schemes are further described in Table 1. The FFF is being implemented and administered by Birmingham City Council (BCC), Solihull Metropolitan Borough Council (SMBC), and the health inequalities unit at BSOL-ICB. Governance is provided by the relevant Place Committees at each council and the Challenge Fund Task Force at BSOL-ICB. Project leads are expected to reserve 15% of the funds they are allocated toward monitoring the take-up and impact of their projects with each lead being offered technical assistance, and training on data collection and interpretation (21, 22).

**Table 1:** Summary of the three categories of FFF funding streams subject to evaluation.

Category of grant	Location	Also known As	Delivery model	Targeted inequalities	Total funding	Allocation to each project (duration)
Small-Scale Grants	Birmingham	Citywide/ Locality Small Grants	Community-developed and led and developed initiatives	Children’s health (“Best start in life”) Healthier lives in underserved communities, Early intervention and health promotion Empowering and connecting communities	£3.18 m	£15 k per year up to a total of £45 K (over 3 years)
	Solihull	Small Grants	Community-developed and led and developed initiatives	Improving population health and healthcare Tackling unequal outcomes and access Enhancing productivity and value Supporting the broader social and economic development of Solihull.	£216 K	£10 K per project (between 12 and 18 months)
Large-Scale Grants	Birmingham	Citywide/Locality Partnership Grants	Two or more organisations working together one must be health and social care	Develop lasting partnerships between health (and social care) organisations and the communities they serve.	£10.22 m	£50 K-500 K (Between 2 and 3 years)
	Solihull	Early Implementer Grants	Two or more organisations working together, one of which must be from the VCFSE sector	Support for Family Hubs, Children and young people’s emotional wellbeing, Heart and lung health.	£1.6 m	Unspecified (Between 2 and 3 years)
Infrastructure Grants	Birmingham/Solihull	Challenge Fund	Led by BSOL-ICS health inequalities group across two phases	Three cross-cutting themes Data analysis, Community engagement, Training in awareness for workforce and community	£3 m across two phases	Unspecified (Between 2 and 3 years)

This protocol describes the independent evaluation of the FFF being led by the University of Birmingham. It follows best practice in the evaluation of large-scale public health programmes reliant on real-world data, by adopting a flexible, phased approach that will produce formative as well as summative findings over the course of the FFF programme (13, 23). This approach is expected to provide iterative learning and opportunity for adjustment for administrators and project leads, ultimately leading to more sustainable impact (13, 23). It will use qualitative and quantitative methods over three inter-linked work packages to gather evidence on the factors that influenced the structure and delivery of the FFF, the support, skills and time needed to create more efficient and effective collaborative partnerships, and the individual and collective impact of all projects on participants, alongside the contextual factors influencing their delivery.

## 2 Methods and analysis

### 2.1 Aims

The overall aim of the evaluation is to determine how and to what extent the FFF has helped improve a variety of health, well-being, and lifestyle factors, amongst underserved populations living in Birmingham and Solihull.

This will be achieved by meeting 3 key objectives:

1. Identifying and describing the decision-making process for the design and delivery of the FFF programme and how this process might be amended or optimised for similar public health programmes in the UK and beyond.
2. To understand how communities, health and social care providers and the Voluntary Community Faith Social Enterprise (VCFSE) sector can be enabled to create sustained collaborate partnerships that deliver lasting improvements to public health.
3. To understand the impact of the programme at a collective level by collating participant data across projects and determining any characteristics or contextual influences shared by successful interventions.

### 2.2 Evaluation design

The evaluation is using mixed methods, consisting of a combination of quantitative data derived from surveys and measurement tools, and qualitative data collected from multiple semi-structured interviews. The work will be conducted across three inter-related work packages corresponding to each of the three objectives outlined above. These are summarised in Table 2 and described in detail below. We cannot predict with certainty the number of individual participants that will complete the survey tools, but the total number of interviews is intended to provide suitably rich data within the logistical constraints of the evaluation (24), and using purposive sampling where practicable (25). The qualitative and quantitative data from the various elements will be combined using a framework-based synthesis (26) informed by the Consolidated Framework of Implementation Research (27). For those projects where their intervention does not readily allow a “before and after” survey measurement, or where the data are not covered by the permissions of this evaluation, the narrative summation and/or aggregated and anonymised data will be described individually and where possible combined in the broader framework analysis.

**Table 2:** Overview of evaluation design.

	Work package 1	Work package 2	Work package 3
Purpose	Factors influencing design and development of FFF	Factors influencing the successful development of inter-organisational partnerships	Individual and collective impact of funded interventions Experiences of participants and intervention leads
Data type	Qualitative	Qualitative	Qualitative and quantitative
Data collection	Semi-structured interviews Document analysis	Semi-structured interviews	Quantitative: Individual participant data, collated by project and type of intervention Qualitative: semi-structured interviews
Data analysis	Directed content analysis using CFIR	Directed content analysis using CFIR	Quantitative: Descriptive statistics and analysis of collated intervention data Qualitative: Directed content analysis using CFIR

The integrity of the evaluation will be supported by regular meetings of the evaluation working group containing representatives of key stakeholders including BCC, SMBH, and the ICB, including where appropriate service leads from NHS organisations and the operational working groups responsible. The early sight of outputs for comment will inform any refinement of the FFF programme.

### 2.3 Theoretical lens

**Figure 1:**
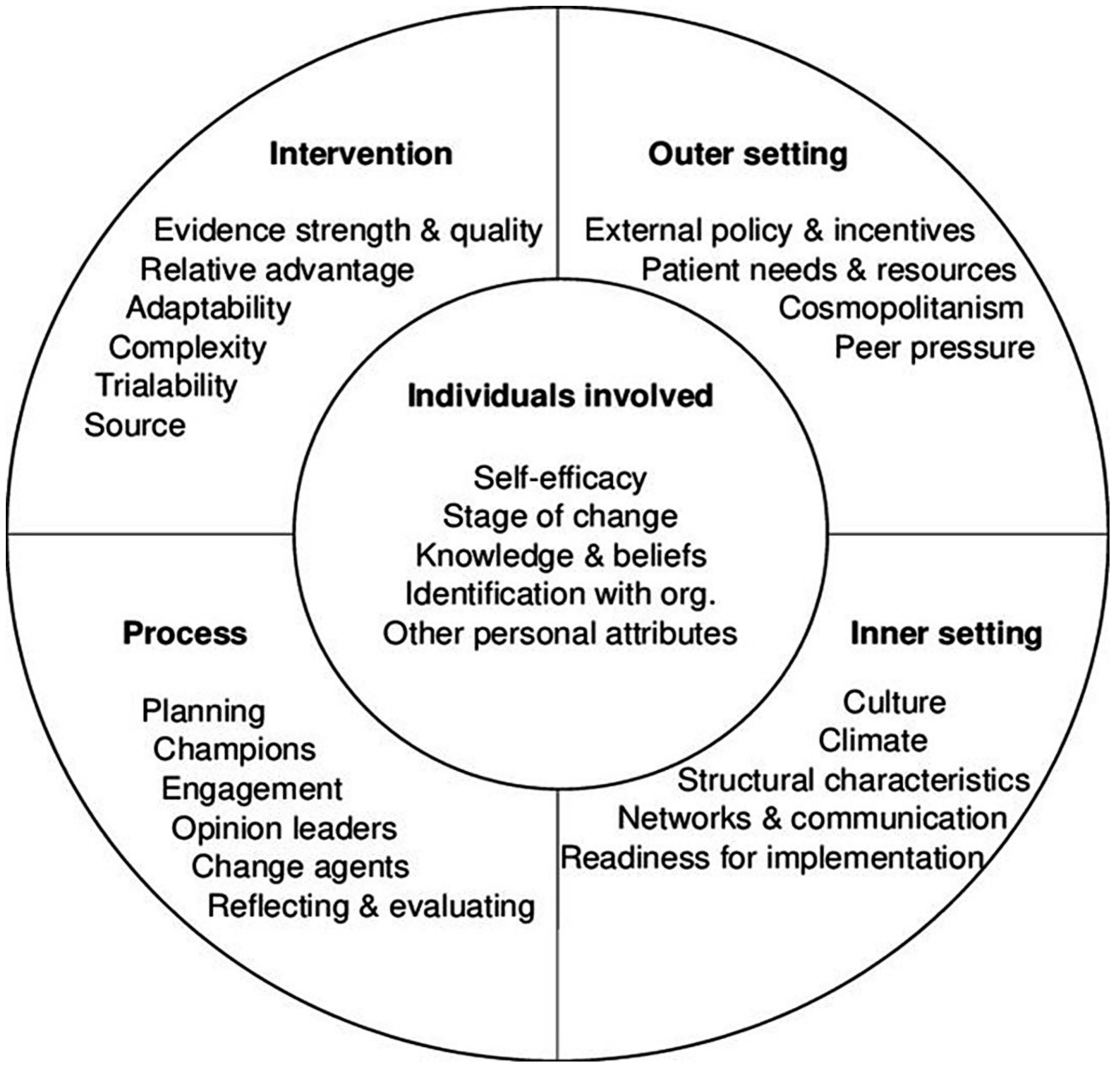
Summary diagram of the five domains and key constructs of the Consolidated framework for implementation research (27, 55).

We will use the C*onsolidated Framework of Implementation Research* (CFIR) to describe and present our findings (27). The CFIR consists of 39 constructs presented within five key domains: (1) *Intervention Characteristics*, relating to the design and development of the intervention; (2) *Outer Setting*, referring to the influence of the environment external to the organisation; (3) *Inner Setting*, describing factors integral to the organisation or organisations involved; (4) *Characteristics of Individuals*, consisting of the knowledge and beliefs of stakeholders; and (5) *Process of Implementation* which entails the planning and management of the implementation of an intervention. These domains are summarised in Figure 1.

The CFIR has been successfully used in the post-hoc deductive analysis of qualitative data (28) and its conceptual clarity has enabled it to capture the complexity of implementation across various health settings (29–35). The analysis will be further supported by Bambra’s typology of public health interventions, i.e., whether they are aimed at strengthening individuals, strengthening communities, improving living and school/work conditions, or promoting broader health policies (36). The CFIR will enable a framework synthesis of the quantitative and qualitative data.

### 2.4 Evaluation overview

#### 2.4.1 Work package 1: a qualitative evaluation of the processes and decision-making around the design and delivery of the Fairer Futures Fund

The structure and plan for the delivery of the FFF was developed by a range of senior stakeholders from across BSOL-ICS including health and social care organisations and local authorities including senior public health practitioners at BCC and SMBC, and members of BSOL ICB. The intention is to gain insight into the operational and organisational factors that influenced the design and delivery plan of the FFF. This includes conversations around target populations, and the nature and content of consultation across BSOL-ICS member organisations. This data will enable reflection on areas of best practice or where improvements might be made to inform future programmes. This work package will explore the development of the FFF in two ways. The first involves conducting a series of semi-structured interviews with the individuals involved in the establishment or oversight of the FFF. The second will corroborate and complement the data gathered from the interviews by analysing any relevant documentation produced in relation to the establishment of the FFF.

#### 2.4.2 Work package 2: a qualitative evaluation of collaborative working and co-production in large-scale grants

This work package aims to understand the entities, processes and structures that underpin sustainable collaborative partnerships and effective co-design and delivery in the context of the projects funded by Large-Scale Grants (overseen by BCC and SMBC) and the cross-cutting Infrastructure Grant (overseen by BSOL-ICB). Ultimately this work will enable the description and comparison of the various approaches taken to engage communities, establish and maintain collaborative partnerships, gathering data on the methods used to promote inclusive co-design and production.

#### 2.4.3 Work package 3: evaluation of health and well-being outcomes and provider and participant experiences across all grants

This work package consists of both quantitative and qualitative elements. The quantitative element consists of collecting data on project characteristics (e.g., health outcome, target population, and location and number of sessions) and on participant outcomes. The data will be collected from projects funded by all three categories of grant, i.e., Small-, Large-, or Infrastructure type grants. This individual level data will then be collated and analysed using these characteristics to provide a quantitative understanding of the overall impact of the various projects across the FFF, and identification of any characteristics shared by successful projects. The quantitative data will be contextualised by a series of semi-structured interviews exploring the experiences of a range of project leads and participants.

### 2.5 Data collection

#### 2.5.1 Work package 1

The topic guide for the semi-structured interviews will cover all elements of the design and development process including who was involved in initial discussions, the use of any evidence-based rationale in the design of the FFF, how the Birmingham and Solihull Integrated Care System’s priorities for health and care were operationalised as funding streams, and how competing motivations from the various member organisations were negotiated and aligned. We will aim to carry out interviews with a purposive sample of up to three staff from each organisation (including staff from BSOL-ICB, BCC and SMBC) as well as VCFSE sector organisations and consultancies to reach a maximum of 25 stakeholder interviews as per the sampling framework (See Supplementary Table S1). Identification and recruitment of relevant individuals, including those who have left or moved to different roles within their organisations, will be supported by the Evaluation Working Group and via existing staff.

Any relevant FFF documentation will be provided or otherwise identified by interviewees to complement the data collected during the interviews. This will include any protocols, evidence syntheses, or minutes of meetings related to the design and development of the FFF programme.

#### 2.5.2 Work package 2

The data collection has two key elements: The first consisting of semi-structured interviews with project leads drawn from a range of health and care organisations, the VCFSE sector and any other relevant stakeholders working to deliver projects funded by Large-Scale Grants across BCC and SMBC. Topic guides will consist of questions around the methods of identification and engagement of various stakeholders, the maturity of existing cross-organisational partnerships, how equitable management of relevant collaborators was managed, and how shared success might be measured or otherwise understood. The second element consists of a series of semi-structured interview conducted with senior stakeholders involved in projects funded by the Infrastructure grant scheme. The topic guide in this instance will attempt to understand the degree to which the projects funded by the Infrastructure grants were able to meet its key objectives, including the criteria and success of funding decisions, the buy-in from across BSOL-ICS to infrastructural changes, and the success with which the workforce was engaged in change.

To complement these semi-structured interviews, we will also be undertaking structured observation of any co-design or co-production meetings used to support the delivery of projects funded by the Infrastructure or Large-Scale grant schemes. These will help us to understand the dynamics and outputs of these meetings including how successfully any potential hierarchical or power imbalances were negotiated.

The work exploring the projects funded by Large-Scale Grants in BCC and SMBC will be conducted across five sites: Four in Birmingham, one where collaborative working (between Health and Social Care organisations and local communities) was established prior to FFF and the second where any such partnerships are new or emerged in response to the FFF, and a further two sites where they are delivering projects funded by two “clinical priority” collaborative grants initiated by health care organisations. The fifth site will be selected in Solihull where they will explore an “early implementer” grant.

We will aim to carry out up to 48 interviews as part of WP2 (See Supplementary Table S2 for sampling framework). The research team will identify the co-design meetings they wish to observe in conjunction with BCC, SMBC, and the organisations involved in delivering the funded projects. There will be a maximum of two co-design meetings observed in each of the five localities, i.e., a minimum of 10 meetings.

For the projects funded by the Infrastructure Grant we will aim to carry out up to 20 semi-structured interviews with a representative sample of stakeholders drawn from projects within each of the three priority areas, i.e., data sharing, community engagement and training in awareness of health equalities (See Supplementary Table S3 for sampling framework).

#### 2.5.3 Work package 3

The data collection in this work package consists of two elements consisting of quantitative data and qualitative data. Quantitative data will be captured from each of the projects funded by the three categories of grants, i.e., Small-Scale Grants, Large-Scale Grants, and Infrastructure Grants. This data collection will be facilitated by REDCap, a secure web application for building and managing online surveys and databases.^1^ Completion of the online surveys will be supported by those delivering the project, and/or made available as a hard copy translated into Easy Read format (37). The surveys will collect data in three domains, specifically:

1. *The key characteristics of each intervention:* These will include their overall aim, target population, mode of delivery, duration, location, and the identity and training of facilitators. Project leads will complete these at the beginning of the project but will be offered the opportunity to update these characteristics every 12 months (where relevant) to reflect any changes in the way the project is delivered.
2. *The demographics of each participant*: The data captured will include age, sex, ethnicity, and religion, in line with best practice and captured once, when they begin the project.
3. *Data on participant outcomes:* This will include data captured through a combination of novel and/or pre-validated survey tools selected by BCC to support consistent data collection across interventions. These tools will be used to consistently explore changes in a range of domains, for example, physical activity (active lives survey), social isolation (3-item loneliness scale), or wellbeing (Warwick-Edinburgh Mental Wellbeing Scale). The intention is that these surveys will be issued over three time points; before starting the intervention, after completing the project (dependent upon the requirements and expectations of the project as not all will have before and after measurements). The third and final survey will be issued 6 weeks after completion of the intervention to provide some understanding of its sustained impact. To encourage participation, project leads will, if more appropriate for their participants, be able to print the survey out and/or enter the data onto REDCap manually. Surveys will be made available in a variety of languages or in ‘Easy Read’ as required.

The qualitative element of Work Package 3 involves conducting semi-structured interviews with a sample of project leads and participants on their experiences of leading or participating in a variety of projects. We will using maximum variation sampling to gain insight into a range of interventions by size, design, intended outcome, and location within Birmingham and Solihull (38). The topic guide will include questions on the elements that supported successful delivery of, or participation in, individual projects. There will be up to 128 interviews in total (See Supplementary Table S4 for sampling framework).

### 2.6 Data analysis

#### 2.6.1 Qualitative data (work packages 1,2 and 3)

The data from the semi-structured interviews from across the three work packages and data from the observed co-production meetings will be used in a directed content analysis to populate the CFIR framework (39, 40). This will enable a structured description of the data describing the design of the FFF, the nature and experience of collaborative partnerships and the data describing the experiences of participants and leads. Including organisational and contextual influences. The findings will support the identification of barriers and facilitators to the development and implementation of FFF, to inform future public health policy initiatives and grant programmes.

#### 2.6.2 Quantitative data (work package 3)

The quantitative survey data will be extracted from REDCap and analysed using SPSS statistical software.^2^ The data collected will be summarised with descriptive statistics and depending on the number of responses received, we will use univariable analyses, e.g., paired t-tests (where pre- and post-intervention outcome data are available) and/or multivariable regression analysis to assess the effectiveness of the FFF activities. An attempt to measure the overall effect of FFF, for example with a target or health outcome, will be completed by pooling data collected using the same survey tools across projects. Using the data captured on the characteristics of the intervention and demographic characteristics of participants, will allow us to further understand the role of individual design components on the outcomes of interventions for example by age, ethnicity, condition, and/or mode of delivery. We will also explore the potential of conducting an intersectional sub-group analysis informed by the data collected on participants characteristics (41). Where appropriate we will use meta-analytic techniques. Effect estimates will be reported with corresponding 95% confidence intervals. The level of missing data will be assessed and imputation analysis undertaken if applicable.

### 2.7 Patient and public involvement

The aims and outcome measures were developed and informed using patient and public health data and by a series of listening exercises which informed BSOL-ICS 10-year health inequalities strategy and the subsequent funding streams of the FFF. The evaluation plan was designed by University of Birmingham in collaboration with BCC, SMBH, BVSC and the communities they represent. University of Birmingham will establish a Patient Public Involvement and Engagement panel to offer feedback throughout the duration of the evaluation including on recruitment materials and topic guides. Ultimately UoB will co-produce dissemination materials with the PPIE panel and other community partners containing interim and final findings.

## 3 Discussion

The FFF program contains a number of key components necessary for developing and delivering a successful public health initiative at scale, including rigorous, real-time monitoring and evaluation, partnerships and coalitions with public- and private-sector organisation, and political commitment accompanied by dedicated resource (42).

The amount invested, the innovative nature of the funding allocation, and the visibility of the FFF programme warrants this detailed and objective evaluation by experienced researchers and evaluators which can help refine future large scale (public) health initiatives and grant programmes. Each Work Package will make a distinct yet complimentary contribution to this broad aim, providing evidence in three key domains. First, to understand more of how the initiative was designed and developed, Second, best practice in collaborative working and co-production across multiple health and care organisations; Third, providing evidence of the overall impact of the funded projects on participants and any shared characteristics of design attributes that influenced the level of success.

Understanding the factors and conversations that shaped the design of the FFF in terms of the aims and specification of each grant stream, and the funding allocated to each will help inform the development of future grant schemes. It’s understood that in many cases the delivery of public health initiatives at scale commonly occur opportunistically, in response to the funding available (43). This means that a framework to support the process informed by the experiences of those that designed the FFF will help future initiatives better identify the needs of governance, leadership, and expertise that should be involved. It may also help determine the reporting structures that need to be in place to ensure accountability for the initial decisions and the range of partners involved in the design process (21, 43).

The FFF is hoping to leave a legacy of collaborative partnerships and co-production activities capable of meeting the evolving needs and expectations of all stakeholders including local underserved populations (5, 44, 45). To understand how that can be effected, including which approaches and initiatives were most effective in facilitating the partnerships envisaged, the evaluation is dedicated to understanding the collaborative processes involved and the experience of all stakeholders. This includes the level of empowerment and training provided for “non-professional” partners, organisational capacity and commitment, and how any hierarchies are flattened in pursuit of open and equitable working (46). In this way we hope to gain more of an understanding of the skills and time needed to create more efficient and effective partnerships, and how active community-level participation can be embedded in future funding initiatives (10).

Finally, we want to understand the summative impact of the various projects funded across all three grant schemes through the use of a pre-determined set of survey tools. The capacity of community grounded initiatives to advance health equity, and contribute diverse benefits to local populations is increasingly understood but quantitative measures of impact at a project level are often hindered by small sample sizes and incomplete data sets (47). Our attempt to collate data across projects is new, but supported by the survey toolkit, a freely available online survey portal that hosts these tools and the data, and by training and dedicated funding being provided to each service lead for the purpose of data capture. We may not successfully meet our aim of providing authoritative quantitative data but the qualitative interviews of participant experiences will tell us more as to if and how such an approach might work in the future.

### 3.1 Limitations and plans to mitigate them

There are some potential risks to the delivery and evaluation of FFF. It is acknowledged that using an online survey system to collect individual participant data risks excluding those that are less digitally active which is true of many of those in underserved populations. This means that questionnaires are more likely to be completed along lines of age, gender and education strata (48). For this reason completion of the online surveys will be supported by those delivering the project, and they will also be made available as a hard copy translated into Easy Read format (37).

It is widely understood that participant recruitment and retention can be particularly challenging where health-related interventions involve underserved or marginalised populations (49). To support engagement and retention the FFF is following best practice by partnering with community entities, using population-appropriate modes of communication and data collection, and by conducting interventions in familiar settings at convenient times (49).

The quality and quantity of the participant level data is also reliant on project leads, including their support in completing surveys with less digitally literate participants. Related to this, the ability to valuably collate any participant level data is dependent upon there being enough projects using the same survey tools. Because of the unknown variation in the number of participants, content, and structure of each project, power calculations are not applicable (50). At the time of writing, the NHS in England is evolving and ICBs are merging and responsibilities and priorities converging (51). However, the funding for the FFF and its allocation has been approved, and ‘ring-fenced’. It is possible that the evolving health and social care system will influence the outcomes of the FFF and though it may not be possible to control for these influences, the qualitative interviews will provide valuable insight into the extent and nature of their impact. Similarly, with the mechanisms and structures for funding allocation, project support, data collection and evaluation agreed and understood by all stakeholders it is expected that the FFF will be suitably protected from major organisational or staffing changes amongst the key stakeholders.

The reliance on self-reported data risks the introduction of bias from sampling, the recall period or selective recall or social-desirability bias, where the respondent tries to provide the responses they believe are expected of them (52). There can also be response-shift bias whereby their frame of reference shifts between time points as a result of participant exposure to an intervention (53). Where possible we will seek to minimise the impact of this bias by using corroborative (qualitative) data, validated survey tools and explicit descriptions of participant characteristics (52, 54).

## 4 Ethics and dissemination

### 4.1 Ethics

This evaluation protocol has been approved by the University of Birmingham Science Technology Engineering and Mathematics ethical review committee as a service evaluation (ERN_3203-Dec2024). If there is a need to access health data, for example in the evaluation of one of the projects funded by the Large-Scale Grants or participant data required from under-18 s, or otherwise vulnerable members of the population, then additional ethical approval will be sought from the Health Research Authority. It is possible that some of the quantitative and qualitative data may be of a potentially sensitive nature dependent upon the intervention. However, the University of Birmingham’s evaluation team has experience in conducting research in similar health and well-being related topics and will ensure the appropriate signposting and safeguarding measures are in place for those participating in the evaluation. We will follow an informed consent process and maintain the anonymity of participants and organisations as per the approvals granted. All data will be managed in line with legal and regulatory requirements, including the General Data Protection Regulation (GDPR) and the Data Protection Act (2018).

Given that the evaluation is being conducted of standard non-invasive health and well-being promotion activities, the occurrence of serious adverse events is not anticipated. In the unlikely event that an incident is reported to an evaluator that is found to be serious, unexpected and possibly linked to an FFF intervention we will report this in line with the sponsor’s safety reporting procedures and/or prepare information about where any such participants can obtain the support that they require. This process is outlined in each Participant Information Sheet.

### 4.2 Dissemination

Dissemination of our interim and summative findings will be facilitated through existing networks within BSOL ICB/ICS, Birmingham and Solihull Councils Place Committees, and the VCFSE sector. There will be at least one peer-reviewed publication relating to each work package. Beyond this dissemination methods are expected to include slide packs and appropriate non-expert forms of dissemination such as videos, blogs, podcasts, and workshops. Throughout the evaluation, we will regularly share feedback on findings through existing networks and through a dedicated FFF website. Interim reports will be submitted annually (December 2025, December 2026 and December 2027) and a final report (June 2028).

## Footnotes

^1^ https://www.project-redcap.org/

^2^ https://www.ibm.com/spss
