# Supplementary material for: Mixed-method evaluation of the Fairer Futures Fund: a £22.2 m public health initiative designed to reduce health inequalities in Birmingham and Solihull (UK)
Source: Front Public Health. 2025 Sep 29;13:1658646. doi: 10.3389/fpubh.2025.1658646 (PMC12515923; doi:10.3389/fpubh.2025.1658646)
Supplement: Supplementary file 1 [file Table_1.DOCX]

***Supplementary File 1***

Table S1. Sampling framework for WP1 interviews with service providers

| ***Participant category*** | ***Number of interviews*** |
| --- | --- |
| Senior stakeholders from clinical organisations | Up to 5 |
| Senior stakeholders from participating councils | Up to 8 (4 per council) |
| VCFSE sector organisations | Up to 8 (2 per organisation) |
| Others (Consultancies or other participating organisations) | Up to 4 |
| **Total** | **Up to 25 interviews** |

Table S2 Sampling framework for WP2a

| ***Participant category*** | ***Number of interviews*** |
| --- | --- |
| Senior stakeholders from clinical organisations | 10-12 |
| Senior stakeholders from Community groups | 10-12 |
| VCFSE umbrella organisations | 10-12 |
| Others (Consultancies or other participating organisations) | 10-12 |
| **Total** | **40-48** |

Table S3 Sampling framework for WP2b

| ***Participant category (Theme)*** | ***Number of interviews*** |
| --- | --- |
| Senior stakeholders (Data analysis) | 4-6 |
| Senior stakeholders (Community engagement) | 4-6 |
| Senior stakeholders (Workforce training) | 4-6 |
| Senior stakeholders (System-wide projects) | 4-6 |
| **Total** | **16-24** |

Table S4 Sampling framework for WP3b

| ***Interviewee category*** | ***Number of interviews*** |
| --- | --- |
| *Small Grant* | |
| Intervention lead/facilitator (by target outcome*) | 10-12 |
| Participant (by target outcome*) | 10-12 |
| Intervention lead/facilitator (by target population*) | 10-12 |
| Participant (by target population*) | 10-12 |
| *Larger Grant (Partnership BCC)* | |
| Intervention lead/facilitator | 20-22 |
| Participant | 20-22 |
| *Larger Grant (Implementer SMBC)* | |
| Intervention lead/facilitator | 4-6 |
| Participant | 4-6 |
| *Larger Grant (Challenge Fund)* | |
| Intervention lead/facilitator | 10-12 |
| Participant | 10-12 |
| **Total** | **108-128** |

*To be confirmed when full scope of projects known
